# Chemistry of the Four Seasons

**Published:** 1847-01

**Authors:** 


Art. II.—*Chemistry of the Four Seasons.* By Thomas Griffiths.—*London,* 1846. pp. 495.

This little volume combines, in an emiment degree, amusement with instruction. It contains a discussion of the chemical, physical, and vital phenomena characteristic of the four grand periods or seasons of the year; of those changes which ever recur with the varying amount of light and heat, depending on the varying obliquity of the solar rays. The author states that the chief object of his essay is to adduce a few of the principal phenomena which admit of explanation and illustration through the medium of chemistry; and he has well executed his task. Commencing with a popular description of those elementary bodies which play an important part in the economy of Nature; which alike form the constituents of a fertile soil, and the atmosphere, of plants, and of animals; he proceeds to the description of the chief phenomena of vegetation in its chemical relations. His statements are illustrated by the details of the more simple experiments on which they are grounded, and these are accompanied by appropriate diagrams. The laws and properties of those wonderful and mysterious agents—heat, light, electricity, galvanism, and magnetism, are appropriately discussed, and their influence on vegetation noticed.

Taken as a whole, we may say with confidence that this volume illustrates in a simple, popular, and amusing manner, the chemical physiology of plants, and completely fulfils the author's aim in writing it, viz., the communicating the more important facts of agricultural chemistry, in an agreeable manner, and unclogged by the deeper investigations and speculations of science. Any person ignorant of the facts on which our knowledge of the chemistry of vegetation is founded, and of the laws which regulate the chemical changes constantly going on in plants, will do well to possess himself of this volume; for by an attentive perusal, aided by a repetition of the experiments there indicated, he will prepare himself for the study of more extended and profound works on the same subjects. We would especially recommend it to youths commencing the study of medicine, both as an incentive to their natural curiosity and an introduction to several of those branches of science which will necessarily soon occupy their attention. We would notice further, and with commendation, that a sound and rational natural theology is spread through the whole work.

